# A New Podcast for Reducing Stigma Against People Living With Complex Mental Health Issues: Co-design Study

**DOI:** 10.2196/44412

**Published:** 2023-05-05

**Authors:** Elise Carrotte, Fincina Hopgood, Michelle Blanchard, Christopher Groot, Lisa Phillips

**Affiliations:** 1 Melbourne School of Psychological Sciences The University of Melbourne Parkville Australia; 2 Anne Deveson Research Centre SANE Carlton Australia; 3 School of Humanities, Arts, and Social Sciences University of New England Armidale Australia

**Keywords:** mental illness stigma, co-design, podcasting, participatory methods, attitude change

## Abstract

**Background:**

*Mental illness stigma* refers to damaging stereotypes and emotional responses around the experience of mental health issues. Media-based interventions have the potential to reduce the public’s stigmatizing attitudes by improving mental health literacy, emotional appeal, and the intimacy of address. As audio-based media facilitating storytelling, podcasts show potential for reducing stigma; however, it is unclear what features could make a podcast effective or engaging.

**Objective:**

The Co-Design and Anti-Stigma Podcast Research (CASPR) study aimed to collaborate with key target audience members to inform the development of a new podcast. This podcast primarily aims to reduce listeners’ stigmatizing attitudes toward people living with complex mental health issues.

**Methods:**

This study was adapted from Experience-Based Co-Design methodology. The first part, *information gathering,* involved a web-based mixed methods survey with 629 Australian podcast listeners to explore their interest and concerns around podcasts. Then, a series of focus groups were held with a purposive sample of 25 participants to explore the potential benefits and challenges of the podcast format. Focus group participants included people with lived experience of complex mental health issues, media and communications professionals, health care professionals, and people interested in workplace mental health. The second part, *co-design,* constituted 3 meetings of a co-design committee with 10 participants drawn from the focus groups to design the podcast using brainstorming and decision-making activities.

**Results:**

Most survey respondents (537/629, 85.3%) indicated a willingness to listen to a podcast about experiences of mental illness stigma; participants indicated preference for semistructured episodes and a mixture of light and serious content. Focus group participants identified potential challenges with appealing to listeners, making the content emotionally resonant and engaging, and translation to listeners’ attitude change. The co-design committee collaborated to achieve consensus on the focus of individual episodes: domains where stigma and discrimination are common, such as workplaces and health care settings; the structure of individual episodes: storyboards that centralize guests with lived experience, featuring explicit discussions around stigma and discrimination; and overarching content principles, including a sincere, empathetic, and hopeful tone; using plain language; having clear calls to action; and providing listener resources.

**Conclusions:**

The co-design process informed a podcast design that features lived experience narratives with an explicit focus on stigma and discrimination, highlighting the realities of stigma while acknowledging progress in the space and how listeners can contribute toward social change. This study allowed for an in-depth discussion around the strengths and limitations of such a podcast according to different target audience members. The co-design committee designed key elements of a podcast that has the potential to minimize the limitations of the format while embracing the benefits of podcast-based storytelling. Once produced, the podcast will be evaluated for its impact on attitude change.

## Introduction

### Background

In co-design, key stakeholders collaborate in a meaningful way to produce an outcome, and people’s lived experiences are centralized and championed [1]. Over the last decade, there has been an increased push for co-design across health care interventions and in the creation and implementation of new services and tools [1-3]. The benefits of co-design and other participatory methods include a redistribution of power, where stakeholders are empowered to have an active role in designing services and tools [1]. Furthermore, the end products of co-design may be better placed to meet the needs of the communities they serve, improve patient experiences, and ultimately increase the likelihood of meaningful change [4].

Researchers have called for more engagement of stakeholders, centering on people with lived experience, in the design and implementation of interventions that aim to reduce *mental illness stigma*: a set of negative and damaging stereotypes and emotional responses around the experience of mental health issues [5,6]. Existing stigma reduction interventions promote a range of messages, predominantly encouraging help-seeking, promoting understanding, reducing the avoidance of diagnostic labels, improving how people living with mental health issues are represented in the media, and encouraging self-worth among people with lived experiences [6]. Such interventions have resulted in short- to medium-term knowledge and attitudinal improvements among the general public, such as increased acceptance of seeking professional mental health treatment [6-9]. Yet, the public generally still hold high levels of stigmatizing beliefs and engage in high levels of discriminatory behaviors toward people living with complex mental health issues (such as schizophrenia, bipolar disorder, personality disorder, eating disorders, and severe depression and anxiety) [6,9]. This indicates that more work is needed to result in long-term improvements, such as trialing new interventions and messaging developed via co-design. Of note, in Australia, only 59% of stigma reduction programs report involving people with lived experience in their design, although 76% report involving people with lived experience in their delivery [10].

### Designing a Successful Stigma Reduction Podcast

Meta-analytic evidence supports the use of audio and video narratives for persuasion in health communication [11]; however, the use of podcasts for health communication is a relatively new area. Podcasts are internet-based audio files typically available as a series of episodes streaming on the web. Approximately 1 in 3 Australian adult podcast listeners have tuned into a mental health–themed podcast in the last year [12]. Podcasts are increasingly being used as an adjunct to counseling and psychological therapies and in psychological research [13] and are being frequently used to share narratives (cohesive personal stories, testimonials, etc) from people with lived experience of mental health issues [14,15]. Sharing personal experiences around social issues in this format may increase listeners’ understanding and empathy [7,16,17], humanizing storytellers and potentially reaching a wide audience [18]*.* Cross-sectional studies have identified associations between listening to mental health–themed podcasts and lower levels of stigmatizing attitudes [12,19], and an experimental study found that listening to a podcast interview with a clinician about psychosis reduced listeners’ endorsement of myths and stereotypes about this diagnosis [20]. Outside the mental health field, research has explored how listening to podcasts can reduce stigma and promote understanding around the themes, such as trauma [21], menopause [22], sexuality and race [23], and domestic violence [24]. Research has also identified how podcast creation and storytelling can be therapeutic processes in themselves; for example, a study described how Indigenous storytelling through podcasts and other modern storytelling outlets can facilitate healing and promote connection with family and culture [25].

However, as an emerging area of study, it is unclear what elements of a podcast might make messaging persuasive and lead to a reduction in stigmatizing attitudes. A Delphi study investigating successful elements of medical education podcasts recommended having a professional tone, ensuring that information is accurate, ensuring that the podcast is accessible, disclosing conflicts of interest, and distinguishing between fact and opinion; more conversational or entertaining approaches were not endorsed [26]. Other studies of medical education podcasts have debated about episode length, with recommendations varying from 10 to 60 minutes per episode [27-29]. In educational settings, student listeners prefer concise episodes and value good sound quality, limited technical issues, and the availability of a transcript [27]. Meanwhile, in the counseling and psychoeducational context, researchers recommend maintaining a professional voice, setting boundaries with listeners, and ensuring that listeners understand what to expect in an episode [13]. It is unknown whether these elements would also apply to podcasts that aim to reduce stigma. Studies describing mental health–themed podcasts and their potential impact on stigma have reported episodes ranging from 15 to 150 minutes [19,20]. Furthermore, in terms of content, the only known experimental study with a stigma outcome involved an interview with a therapist but no interview guests with lived experience [20]; the impact of listening to lived experience stories in this context is currently unclear.

Although some previous studies have described consultancy or participatory approaches to podcast development (eg, Mitchell et al [30], Blum et al [31], and Smith et al [32]), they provide little information about specific, formative podcast co-design processes or decision-making, nor are they specific to stigma reduction. Hence, there is a need to work with members of the target audience for a mental health–themed podcast to understand their needs and preferences through methodologies such as co-design and how to make such a podcast persuasive. The Co-Design and Anti-Stigma Podcast Research (CASPR) study aimed to collaborate with key target audience members to inform the development of a new podcast through co-design. This podcast primarily aims to reduce listeners’ stigmatizing attitudes toward people living with complex mental health issues.

### Overall Design

Although a variety of co-design methodologies have been described for digital health interventions [33], no known studies have detailed such methodologies for developing podcasts. This mixed methods study was adapted from Experience-Based Co-Design (EBCD), a participatory design methodology used to improve health care services, which has been adapted to various mental health care services in Australia [34,35]. EBCD, at its core, involves 2 parts: an exploratory data and information gathering stage and a solution-focused co-design stage [36]. The study was informed by various co-design guidelines and recommendations such as emphasizing respect, informed consent, diversity, accessibility, and meaningful involvement (eg, Slattery et al [4] and Dimopoulos-Bick et al [37]).

Overall, this study focused on key target audiences. Research has identified several domains associated with very high levels of stigma and discrimination in Australia, especially health care settings, workplaces, and media [9]. Hence, the researchers were interested in targeting the podcast toward people working in these 3 domains. Furthermore, people with lived experience were also included. Although the podcast would not necessarily be targeting people with lived experience, who may have lower levels of stigmatizing attitudes than the general population, the podcast would pertain to their personal experiences. Hence, it was assumed that people with lived experience would be invested in this topic and interested in listening to a podcast about the experiences of their peers. Thus, the four key population groups across the study were as follows:

1. People with lived experience of complex mental health issues
2. Health care and mental health care professionals
3. Journalists, media, and communications professionals
4. Managers, human resources professionals, and others with an interest in workplace mental health

The study occurred from January to November 2021. There were 2 parts to the study. Part 1—*information gathering—*first involved a web-based mixed methods survey with general podcast listeners to describe existing podcast use and behaviors and begin to understand potential concerns for the new podcast (part 1.1). After these data were analyzed, a series of 4 focus groups were held (part 1.2) with each individual target audience, deepening the understanding of areas that mattered to each audience. Part 2—*co-design—*involved the establishment of a co-design committee consisting of a mixed group of participants from part 1.2. The methodology and results of each part are presented in order owing to the iterative nature of the study, followed by a general discussion.

## Part 1.1: Information Gathering Survey

### Methods

#### Design and Objectives

This cross-sectional web-based survey aimed to identify podcast listeners’ preferences for and attitudes toward a new podcast on the topic of stigma experienced by people living with complex mental health issues. Questions on these areas were incorporated into a larger survey about podcast listening and mental health; a detailed methodology is presented in a separate publication [12].

#### Participants and Recruitment

The survey involved a convenience sample comprising both general community members, who were recruited primarily through social media, and first-year psychology students, who were recruited through the University of Melbourne’s Research Experience Program. In brief, all participants were required to be aged >18 years old, be living in Australia, and have listened to at least 1 podcast episode in the last 12 months.

#### Ethics Approval

This study was approved by the University of Melbourne Human Research Ethics Committee (2020-20331-13253-3). The participants viewed a plain language statement and completed a consent form before proceeding with the study questions. The first-year psychology students received study credit, whereas others could opt into a prize draw to win 1 of 5 Aus $50 (US $38.50) digital vouchers. All data were deidentified before analysis.

#### Measures and Procedure

The survey was hosted on Qualtrics (Qualtrics International Inc) and open for 4 months (January 2021 to May 2021). The participants provided demographic details, including year of birth, gender, postcode, ancestry, areas of employment or study, and experience of mental health issues. Questions addressed existing podcast listening behaviors and attitudes, and questionnaires on stigma, knowledge, and socially desirable responding style were incorporated; refer to the previous publication for these data [12]. The following questions are the focus of this publication.

First, they were asked, “Would you consider listening to a new podcast on the topic of mental health?” and given the options “yes,” “no,” or “maybe.” Then, they were asked, “Would you consider listening to a new podcast on the topic of stigma experienced by people living with mental health issues?” and given the same response options. No further elaboration was provided to assess the participants’ responses to this language and the framing of the hypothetical podcast topic.

The participants who selected “yes” or “maybe” to the second question were asked to nominate a preference for each of 6 different features of such a podcast: number of episodes, episode length, humor, tone, structure, and guests. Three different options were presented as potential responses to each of the 6 features. The participants could then respond to optional open-text questions: “Why did you say yes/maybe/no to the above questions?” and “Do you have any other comments about your preferences for podcasts on the topic of mental health?”

#### Data Analysis

Quantitative data were analyzed using SPSS (version 25, IBM Corp). Descriptive statistics were generated for all quantitative variables. Open-text responses were reviewed by the lead author (EC) via an informal analysis of common themes using Microsoft Excel (Microsoft Corp). This involved careful reading and grouping of the open-text responses to identify recurring themes associated with strong emotional language from the participants. This was done to identify draft *touch points*—interaction points with the potential podcast that are emotionally resonant [35,37]. Most EBCD-based studies involve the identification of touch points through the analysis of experience data, although studies report a wide range of analytical approaches [36]. After the touch points were drafted, a set of neutral statements associated with the touch points were drafted [35,37]. These statements were then used for prompting group discussion in part 1.2, exploring each touch point in depth [36]. The touch points were later refined in part 1.2 using a formal thematic analysis to inform part 2 (refer to the subsequent section).

### Results

#### Overview

In total, 629 participants completed the survey. In brief, the mean age of the participants was 28.6 (SD 11.4) years; 71.1% (447/629) were women; and 65% (409/629) reported Australian, European, North American, or New Zealand ancestry. Over one-third (250/629, 39.7%) of the participants were first-year psychology students, with the remainder from the general community. Refer to the previous publication for detailed demographic data [12].

#### Willingness to Listen

**Figure 1:**
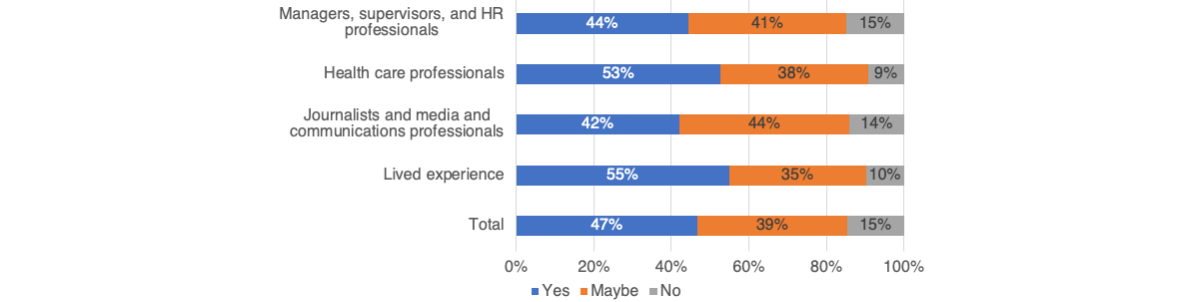
Percentage of participants who would consider listening to a new podcast on the topic of mental illness stigma. HR: human resources.

When the participants were asked whether they would consider listening to a new podcast on the topic of mental health, 56.3% (354/629) said “yes,” 36.4% (229/629) said “maybe,” and 7.3% (46/629) said “no.” When asked whether they would consider listening to a new podcast on the topic of stigma, a slightly smaller percentage agreed; in total, 46.7% (294/629) said “yes,” 38.6% (243/629) said “maybe,” and 14.6% (92/629) said “no.” When stratified by target audience, the participants with lived experience of mental health issues and health care professionals were most likely to say “yes” or “maybe” (Figure 1).

#### Preferences

The participants’ preferences for each of the 6 aspects of the podcast (number of episodes, episode length, humor, tone, structure, and guests) are presented in Multimedia Appendix 1. The participants preferred the “middle” option for each aspect, showing a preference for a mixture of both celebrity and everyday guests, semistructured episodes, a mix of casual and educational tone, a mix of lighter and more serious tone, a length between 10 and 45 minutes, and between 3 and 10 episodes.

In the open-text responses, many participants described factors that would make them likely to listen, including care taken with emotional content, high-quality audio, professional production, and diverse content. On the basis of these responses, 3 touch points and 5 related statements were drafted (Table 1). The touch points represented a roughly linear interaction with the podcast, from whether the people decide to listen in the first place (*marketing and framing*) to the listening experience (*listening to and engaging with episodes)* to the impact of listening (*translation to attitude change and action).*

Regarding the decision to listen, several participants commented that such a podcast would not be relevant or not relatable to them, and a few participants commented on their interest or willingness to reflect on their own prejudices or change their own behavior. One of the participants reflected, “I hesitate to say that most of the general public aren’t interested in educating themselves about discrimination” (female participant, age 52 years). Meanwhile, many participants, especially health care professionals, commented on not having time to listen to a mental health–themed podcast or being unwilling to listen to a mental health–themed podcast in their personal time if they perceived it to be heavy or draining or if they were “already exposed to this information through [their] work” (female participant, age 32 years). Furthermore, the participants were wary that this topic could be depressing or induce feelings of anger or resentment. Many listened to podcasts for entertainment or relaxation and were unsure about listening to what they suspected would be a serious topic, even if they believed that it was important. They also felt that it could be upsetting, particularly for listeners with lived experience, for example, “I live it and experience it. I like podcasts to be an escape, not a trigger” (male participant, age 37 years).

However, many participants commented on their interest in the podcast and the potential for the podcast to be impactful. They commented on their interest in listening to multiple perspectives and the need to ensure that a range of experiences is represented in this format, including those involving complex and severe mental health issues and underrepresented demographics, for example, “I also appreciate podcasts that take into account LGBTQI+ and racially diverse perspectives on mental health” (female participant, age 23 years). Although many participants were interested in others’ personal experiences of stigma and discrimination and believed that sharing these experiences and listening to these stories could be powerful and impactful, some felt that certain podcast structures could be more appealing than others, for example, “Really depends if it will be educational (listen) or just a narrative (not interested)” (male participant, age 44 years).

**Table 1:** Draft touch points and statements generated from the open-text responses in part 1.1.

Touch point	Description	Statement for prompting focus group discussion in part 1.2
Marketing and framing	How the podcast is described and marketed, informing the decision to listen	1. People may not listen if they do not think it is relevant to them—despite holding stigmatizing attitudes themselves. 2. People working demanding jobs may not wish to listen to a podcast on a serious topic.
Listening to and engaging with episodes	The listening experience, emotional resonance, and the messaging of episodes	3. A podcast about stigma and discrimination could be emotional for listeners. 4. It is difficult to represent everyone’s story in a podcast.
Translation to attitude change and action	The impact of the podcast on attitudes and behaviors after listening	5. Telling real stories may (or may not) be enough to change listeners’ attitudes.

## Part 1.2: Information Gathering Focus Groups

### Methods

#### Design and Objectives

Part 1.2 was a qualitative study. It aimed to identify target audience members’ needs and preferences with regard to the proposed podcast, specifically exploring and refining touch points from part 1.1, which would need to be workshopped before the podcast is produced.

#### Participants and Recruitment

A purposive sample of Australian adult podcast listeners was recruited. Each focus group was aligned with 1 of the key target audiences, allowing different participants to explore issues in a homogenous group to maximize cooperation and minimize power dynamics or conflicting views [4,38]:

1. Focus group 1 consisted of SANE’s lived experience peer ambassadors. These participants had lived experience of complex mental health issues and were affiliated with SANE across a range of volunteer and paid roles. All peer ambassadors have previously been trained around advocacy and media engagements, safe storytelling, and maintaining their well-being.
2. Focus group 2 consisted of media and communications professionals (eg, journalists, content writers, and podcast producers)
3. Focus group 3 consisted of workplace mental health (eg, workplace mental health champions, employers, managers, and human resources professionals)
4. Focus group 4 consisted of health care and mental health care professionals (eg, general practitioners, psychologists, and counselors)

The study aimed to recruit up to 32 participants (a maximum of 8 participants per focus group). With the addition of 2 facilitators, 10 people per session was deemed to be an appropriate size to allow for small group activities while not being so large that voices would be missed in larger discussions. Recruitment occurred through flyers and links distributed by mental health, media, educational, or health care organizations through email, newsletters, or social media. Peer ambassadors were invited via direct contact through SANE.

All interested participants conveyed their expressions of interest via the web and completed a web-based consent form via Qualtrics. They provided brief demographic information, including age, gender, ethnicity (optional), and identification with target audiences, and were asked to optionally provide an emergency contact and a wellness plan. The lead researcher (EC) read all expressions of interest and contacted the participants via phone or email. The participants were invited with the aim of maximizing demographic and experience-related diversity, including a range of genders, ages, and ethnicities.

#### Ethics Approval

This study was approved by the University of Melbourne Human Research Ethics Committee (2021-21844-18694-3). On the expression of interest page, the participants viewed a plain language statement and completed a consent form before proceeding. The peer ambassadors were paid an honorarium of Aus $120 (US $90) per focus group in line with SANE’s paid participation policy; other participants were reimbursed with a digital voucher worth Aus $50 (US $37.8). All transcripts were deidentified during the analysis, with names and identifying details removed.

#### Procedure

Part 1.2 focus groups occurred over a 2-week period in July 2021 via Zoom (Zoom Video Communications, Inc). Web-based focus groups were held because of the COVID-19 restrictions at the time. The co-design activities were adapted to the web-based environment [39]. This involved the use of Zoom’s breakout room features to facilitate small group discussions. The website Mural was also used; Mural provides a range of web-based collaborative tools and templates, similar to a digital whiteboard, where participants can create web-based sticky notes. Each focus group was facilitated by the lead researcher (EC), a female psychologist and mental health researcher with experience in focus group facilitation. This study is part of EC’s PhD studies. The lead facilitator aimed to allow for an open exploration of the touch points while grounding the discussion around the limitations of the proposed podcast, such as financial constraints and timelines. There was also a rotating support facilitator with a background in counseling or psychology. The support facilitator was briefed by the lead facilitator in advance and provided practical support with activities, such as taking notes, but was also available should the participants need a break or support. The participants were emailed Zoom links and information about how to use Mural in advance.

Each focus group was audio recorded and lasted for approximately 2.5 hours. Agendas are presented in Multimedia Appendix 2. After an introduction (including a discussion of project background), a discussion of group guidelines, and an ice breaker activity [2], the participants were presented with 3 to 4 statements from part 1.1 (Table 1) in turn to prompt discussion [40]. In small groups, the participants were then asked to complete an “empathy map” activity, with 4 quadrants prompting ideas for what potential listeners of the new podcast might “feel,” “think,” “say,” and “do.” The facilitators shared their screens and typed directly on a Mural template using web-based sticky notes. The participants could use Zoom’s chat features to facilitate conversation [40]. After each focus group, the participants were invited to complete a web-based feedback survey, and if they wished, they could opt in to receive an invitation to part 2—co-design.

#### Data Analysis

The focus groups were audio recorded via Zoom and transcribed by the lead researcher using the transcription software Descript (Descript, Inc). Empathy maps were exported as PDFs. Similar to other EBCD studies [35,41], a thematic analysis was conducted using the methodology outlined by Braun and Clarke [42]. This approach was chosen because it is well established, flexible, and suitable for a largely deductive analysis, such as an analysis for refining the touch points identified in the earlier stages of the study. Two female researchers (EC and a research assistant) with a background in psychological research and counseling analyzed the data. They first familiarized themselves with the transcripts and then coded an extract of the data separately in NVivo (QSR International). The researchers then generated initial codes and together developed an overarching draft framework to consolidate the codes and reflect on their relationships with the touch points. The lead author then coded all the transcripts using these codes, consulting with the research assistant about minor suggested changes to the codes as the analysis progressed and taking down notes and reflections throughout the process. The overarching question informing the thematic analysis was *how do we understand and conceptualize the touch points that are most important to the potential listeners of the podcast?* This was a reflexive process, as the researchers were interested in how those with lived experience of complex mental health issues would interact with and emotionally respond to a new podcast on a challenging topic while recognizing the perspectives of other target audience members who do not have lived experience but may have an interest in mental health from a different perspective. Attention was paid to emotions, topics, and areas that the participants reported feeling the most strongly about and ideas that the participants kept returning to or found divisive. The lead researcher aimed to be mindful of the influence that her training in clinical psychology and familiarity with the medical model could have on her interpretation of the data, for example, the risk of unwittingly prioritizing the views of health care professionals over those of the participants with lived experience.

The feedback survey data were analyzed using SPSS, and descriptive statistics were generated. The open-ended responses were examined, and suggestions for improvement were incorporated into future focus groups, where appropriate. The feedback data are summarized in Multimedia Appendix 3.

### Results

#### Overview

There were 38 expressions of interest across the 4 groups for a maximum of 32 spots. The peer ambassador group had expressions of interest from 14 individuals; 8 (57%) of them were invited, who reflected a range of ages, ethnicities, genders, and lived experiences. Owing to fewer expressions of interest across the other 3 groups, all who expressed interest were invited to join these focus groups, although some attendees could not attend owing to unavailability or illness. In total, 25 participants attended the focus groups: 8 (32%) attended the peer ambassador group, 6 (24%) attended the health care professional group, 6 (24%) attended the workplace mental health group, and 5 (20%) attended the media and communications group. The participants were aged 23 to 62 (mean 37.8, SD 11.9) years. Of the 25 participants, 17 (68%) were women, 7 (28%) were men, and 1 (4%) identified as a trans man and nonbinary. The demographics are presented in Table 2. The analysis explored and refined the 3 touch points first identified in part 1.1 (Table 1).

**Table 2:** Demographics of the focus group participants in parts 1.2 and 2.

		Part 1.2: information gathering (n=25)	Part 2: co-design committee (n=10)
Age (years), range		23-62	25-56
**Gender, n (%)**			
	Women	17 (68)	6 (60)
	Men	7 (28)	4 (40)
	Trans men and nonbinary	1 (4)	0 (0)
**Ethnicity^a^, n (%)**			
	Australian, European, North American, or New Zealander	13 (52)	5 (50)
	Aboriginal	1 (4)	0 (0)
	Lebanese	1 (4)	1 (10)
	Indonesian	1 (4)	1 (10)
	Malaysian	1 (4)	0 (0)
	Indian	1 (4)	0 (0)
	Missing or no response	7 (28)	3 (30)
**Mental health experience^b^, n (%)**			
	A person who experiences distress, trauma, or a mental health issue	17 (68)	4 (40)
	A carer of a person who experiences distress, trauma, or a mental health issue	8 (32)	4 (40)
	Neither	4 (16)	2 (20)
**Target audience identification^b^, n (%)**			
	Peer ambassador^c^	9 (36)	3 (30)
	Health care or mental health care professional	14 (56)	3 (30)
	Journalist, media, or communications professional	5 (20)	3 (30)
	Manager, human resources professional, or a person with interest in workplace mental health	8 (32)	5 (50)

^a^Optional.

^b^Multiple selections possible.

^c^1 peer ambassador attended the media and communications focus group in part 1.2 owing to their availability and experience.

#### Touch Point 1: Marketing and Framing

The participants emphasized that for the podcast to reach people and ultimately make a difference, people must be willing to listen to it. They identified that the core focus of the podcast—real stories from people with lived experience—would need to be appealing to many listeners. They noted that strategic decisions must be made about whose stories to include, including deciding which of a diverse range of storytellers to increase relatability and contextualizing stories rather than just providing storytelling alone:

This is mental health, and it’s stigma, but at the root of it, it’s stories, it’s personal stories. It’s lived experience and that’s what people are resonating with. And that’s what connects. And it’s kind of from that, the behaviors, the awareness, the behavior change kind of gets a jumping off.

Media and communications focus group participant, female, age 36 years

Importantly, some felt that framing the podcast around stigma would prevent many listeners from tuning in. They felt that people with the most extreme stigmatizing views were unlikely to be reached if such framing was used. The participants expected that listeners will already be invested—they may understand the prevalence and impact of stigma and discrimination and already care about the topic and are likely to be well intentioned. Hence, the participants felt that there was a risk of “preaching to the choir.” However, they felt that listeners—even if already invested to a degree—could still learn something from the podcast and find it valuable. Health care professionals also highlighted that the podcast could be used for professional development purposes.

The participants spoke about the importance of accessibility: balancing the episode length with the content; avoiding language that is jargon heavy, highly academic, or clinical; and making transcripts available. They also underlined the importance of production quality; poor-quality audio could be distracting, even causing listeners to turn off in the middle of an episode.

#### Touch Point 2: Listening to and Engaging With Episodes

The participants identified that storytelling techniques and effective messaging can make the listening experience impactful. They universally agreed that listeners *should* feel emotional while listening. They noted that real stories—especially if guests share stories about trauma or stigma and discrimination—are likely to elicit strong emotions such as anger, sadness, and even guilt in listeners, although these stories should not be distressing or triggering. The participants highlighted the role of listeners’ empathy, believing it to be critical for listeners to internalize any messaging. Again, the participants agreed that it was important to include a sense of hope and optimism; otherwise, listeners could feel disempowered and pessimistic:

[With] real stories, they can relate to it more, to the human face to it...it’s not just an abstract thing that they see like a statistic or TV or like, things that they cannot really see...it’s actually a human person, undergoing real emotions.

Workplace mental health focus group participant, male, age 29 years

Views were mixed around which messages would be most effective. Some, particularly peer ambassadors, felt that emphasizing the harsh realities of structural discrimination was important to raise awareness and motivate listeners to change their behavior. Others felt that certain types of messaging could increase listeners’ investment, such as those exploring the economic impact of discrimination or providing recommendations in certain contexts (eg, legal responsibilities in workplaces):

It would be nice to have a podcast that is actually going to deep dive into some of the real issues of trauma as to what is happening in the world and why people struggle.

Lived experience focus group participant, female, age 42 years

#### Touch Point 3: Translation to Attitude Change and Action

**Figure 2:**
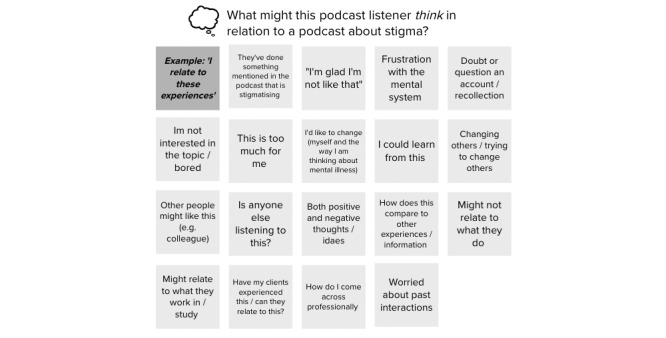
Section of the empathy map from health care professional focus group.

The participants felt that, aside from just raising awareness about stigma and discrimination, the podcast should provide “calls to action ” that motivate listeners to reflect and make positive changes. Suggestions can be both explicit and subtle. Using the empathy map (refer to Figure 2 for example), the participants noted that listeners could begin to challenge their own assumptions; start conversations with family, friends, and colleagues; review legal rights and responsibilities; seek training; and involve clients and patients more in decision-making or treatment processes.

The participants emphasized that listeners need safe environments and resources to make real changes. Not all listeners will be in a position of power where they can influence their environment. The participants also felt that listeners should not feel burdened to somehow prompt dramatic action to change structures or processes and, therefore, feel “disillusioned about what change can be done...powerless” (health care professional focus group participant, female, age 37 years):

Hopefully the listener feels that they have a role to play...hopefully that listener would feel empowered.

Media and Communications focus group participant, female, age 25 years

## Part 2: Co-design

### Methods

#### Design and Objective

Using co-design processes, this part of the study aimed to collaborate with target audience members to inform key elements of the podcast.

#### Participants and Recruitment

After part 1.2, the lead researcher (EC) reviewed 20 expressions of interest in joining the co-design committee. Members were selected to represent a range of demographics and life experiences. It was decided that 10 participants would be invited to become co-design committee members, so that at least 2 members would represent each target audience. The final group size was 12, including the 2 facilitators. All the members were informed of the expected time commitment to encourage consistent involvement, although they could remain on the committee even if they missed a focus group.

#### Ethics Approval

This study was approved through the same application that was submitted in part 1.2 by the University of Melbourne Human Research Ethics Committee (2021-21844-18694-3). The committee members viewed a plain language statement and completed a consent form. All the members were provided with an honorarium of Aus $120 (US $90) per focus group attended. All the transcripts were deidentified, with names and identifying details removed.

#### Procedure

Co-design followed procedures similar to those followed in part 1.1. The committee members attended 3 iterative focus groups, each of which lasted for approximately 2.5 hours and was spaced a fortnight apart from October to November 2021 outside of business hours. A web-based Slack (Slack Technologies, LLC) discussion, described subsequently, continued to be “live” for 2 weeks after the final focus group and was then archived. The total length of part 2 was 6 weeks.

Three key aspects of the podcast were selected for refinement through co-design. These were discussed in the following order, a top-down structure. Each of these 3 areas is related to one or more of the touch points refined in part 1.2.

1. Focus of individual podcast episodes
2. Episode structure (storyboarding)
3. Content principles

Background information was provided through a prereading activity and within the focus groups themselves. The focus groups were facilitated by the lead researcher (EC) and 1 male support facilitator, a counselor and researcher, who attended all 3 groups. The first focus group devoted considerable time to introductions, what co-design involves, the scope and limitations of the study, and establishing group guidelines. The members were informed that the podcast production would most likely involve up to 6 episodes, each lasting for 15 to 30 minutes, owing to resourcing constraints. The rest of focus group 1 and subsequent focus groups involved icebreakers, guided discussions with open-ended questions, brainstorming, prioritization activities, and decision-making. Ideas were iterated in between the focus groups and presented for feedback. At the end of the final focus group, time was taken for a verbal reflection and celebration of what the group had achieved.

The members were given the opportunity to provide feedback via a brief Qualtrics survey after each focus group. Again, feedback was incorporated, where possible, into the upcoming focus groups. More information is presented in Multimedia Appendix 4 (co-design focus group agendas) and Multimedia Appendix 5 (co-design committee feedback). The co-design committee members were updated in between workshops on progress and decision-making and invited to discuss the project through a private Slack workspace. Slack is a web-based discussion application designed for team-based communication. Previous researchers have highlighted the value of unplanned conversations held outside formal co-design activities and using web-based discussion spaces to communicate with stakeholders [2,40], whereas others have discussed the value of collecting data using creative methods such as visual diaries [38]. Slack allowed a space for quieter members and members who may have missed a workshop or part of a workshop to contribute and allowed creative discussions that may take multiple forms of modern, digital communication, such as instant messaging, image-based communication (gifs, images or memes, and emojis), and links to other media.

#### Data Analysis

The focus groups were audio recorded and transcribed as in part 1.1. Mural activities and Slack group discussions were exported via PDFs. As the focus groups involved direct decision-making, no formal thematic analysis was conducted on the transcripts, although they were reviewed to summarize decision-making and identify quotes that represented key decisions. Descriptive statistics from the feedback surveys are presented in Multimedia Appendix 5.

### Results

#### Overview

A total of 10 members joined the co-design committee. One of the members withdrew before the first co-design focus group, so another member was invited instead and accepted. The age of the members ranged from 25 to 56 (mean 39.9, SD 11.3) years. Of the 10 members, 6 (60%) identified as women, and 4 (40%) identified as men. The demographics are presented in Table 2. The first focus group had full attendance (n=10, 100%), 9 (90%) attended the second focus group, and 6 (60%) attended the final focus group. The key decisions made during the focus groups are summarized in Table 3.

**Table 3:** Co-design committee key decisions.

Area	Decision
Episode focus	Each episode will focus on an area (or “life domain”) where stigma and discrimination are known to occur, for example, employment, health care services, mental health care services, media, and educational settings.
Storyboard	Episodes will start with introducing the topic and guests and content warnings. Guests will discuss personal experiences with stigma and discrimination in the relevant setting. Other guests will speak about relevant research, trends, or initiatives. Guests with lived experience will reflect on their experiences. Episodes will end with practical calls to action, resource suggestions, and credits.
Guests	Each episode will feature ≥1 guests who have lived experience of complex mental health issues and come from diverse backgrounds. Academics, lived experience advocates, clinicians, lawyers, and other guests will also be included to provide a “bigger picture” understanding of the topic.
Tone	An overall sincere, empathetic, and hopeful tone will be conveyed through careful use of language and music.
Language	Mostly casual or informal and conversational language will be used. Any academic or conceptual ideas will be kept to a minimum but explained in plain language. The use of “medical model” language and related terminology will also be kept to a minimum.
Messages	Messages will aim to challenge stigma at the individual (1:1) level—not only challenging listeners’ stigmatizing attitudes toward people living with complex mental health issues but also encouraging behavior change. Broader messages around systematic and structural change will be included, but the onus will not be on the listener to solve these problems—instead messages can encourage small steps within the listener’s control. Example messages are as follows: “We all have a role to play in reducing stigma and discrimination, including reflecting on our own attitudes” and “Resources are available in areas like relationships, workplaces, and media that can educate and empower people to reduce stigma and discrimination”
Calls to action	Calls to action will be aimed at the 1:1 level, focusing on practical things listeners can do within their own circles. Example calls to action are as follows: “Share this episode with a friend or family member,” “Check out language guidelines about how to speak about mental health issues,” and “Look at your workplace’s flexible work policies”
Episode titles and descriptions	Episode titles will consist of a few words or a short sentence, clearly conveying the topic of each episode, and be neutral or optimistic in tone. Episode descriptions will be succinct and will focus on *how to improve* outcomes for people living with mental health issues in each context but will not shy away from mentioning terms such as “stigma” and “discrimination.”
Graphic design	Logos are to be simple and uncluttered, focusing on text rather than images.
Accessibility	Episodes made public for streaming will be accompanied by transcriptions.

#### Focus of Podcast Episodes

The co-design committee initially workshopped the focus of individual episodes. The committee was presented with five distinct options for each episode’s focus: (1) an individual’s experience living with mental health issues and recovery, (2) an individual’s experience with stigma and discrimination, (3) one individual diagnosis per episode, (4) a life domain where stigma and discrimination are known to be common, and (5) concepts relating to stigma and discrimination (such as self-stigma or public stigma).

In small groups, members completed a Mural activity involving placing sticky notes against prompts around the strengths and limitations of each option, who would be likely to listen, potential messages, and emotional impact. Representatives then presented the potential advantages and disadvantages of each option to the main group. In brief, the members felt that focusing on stigma concepts could attract an academic audience. Concerns were raised, including generalizability, as the members felt that focusing on individual stories may be unrepresentative or result in listeners struggling to generalize experiences (particularly if stigma is not explicitly discussed). They also felt that listeners may disengage or “tune out” if they disliked the featured guest or struggled to relate to their experience. However, a diagnostic focus can be too medicalized and unintentionally perpetuate stigma:

I don’t think we can ever hope to capture all of everyone’s stories or even all of one person’s story. Everyone has so much to tell.

Committee member, male, aged 56 years

As consensus was not reached in the initial focus group, the discussion continued into Slack. The members raised the idea of combining different options, sharing examples of podcasts that used different voices and structures. Some members commented on using the strengths of each option to strategically counterbalance some of the risks.

Between the focus groups, the lead facilitator (EC) reflected on the comments that challenged assumptions about the need to have a central focus for each episode. She reviewed discussions and iterated the original options, creating 2 new hybrid options for each episode’s focus. Option 1 was an *individual lived experience focus*, where one person’s story would be centralized per episode. Option 2 was a *stigma context focus*, where each episode would focus on an area or domain where stigma is known to occur. Unlike the original options, both new options included an emphasis on lived experience while also acknowledging that other voices may be included (eg, those of clinicians, academics, and advocates) to expand on ideas.

These new options were received positively by the committee, with the members feeling that both options “could work wonderfully.” Some members were drawn to the individual lived experience focus, believing that “the hook [of the episode] is the person” and engaging on a deeper level with the guest and their story. Others preferred the stigma context focus, believing that this could be more persuasive and potentially “more relevant to a much wider audience.”

The committee completed a Mural prioritization matrix exercise, ranking each option on a graph in terms of how engaging or impactful it could be; however, both options were rated highly. Eventually, the decision was put to a vote via an anonymous Zoom poll. Option 2, the *stigma context* focus, won, with 6 (67%) of the 9 attendees present at the time of the vote choosing this option.

#### Storyboard

Once the episode focus was chosen, the committee workshopped storyboards for individual episodes. After discussing storytelling structures and different podcast formats (eg, interviews, panel discussions, and narration), the committee created draft storyboards via Mural in small groups. The Mural activity involved adding sticky notes to “scenes” of the podcast episode, from setup or introduction to resolution or wrap up. The storyboards were presented back to the main group. Common themes included centralizing guests with lived experience but including other guests and including practical calls to action—suggestions for what the listener can do during or after the episode, such as reflection exercises or sharing with colleagues. The members felt that there was a need to “zoom out” and provide more context around trends or research around stigma, if this did not pull focus from the guest with lived experience. The discussion continued in Slack and focused on power imbalances and other issues that occur in advocacy spaces:

I think that it might be nice to have some episodes where there aren’t professionals coming in after [guests with lived experience] because we get that a lot and it is almost like a coming over on top, like, “here’s the real knowledge.”

Committee member, female, age 42 years

**Figure 3:**
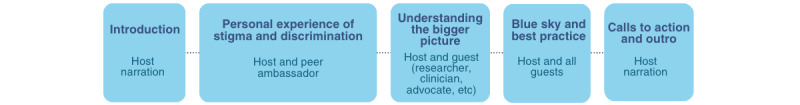
Simplified podcast episode storyboard.

In between focus groups, the lead facilitator created a new, combined storyboard that would be applied flexibly to episodes. This was presented back to the committee members in focus group 3, who provided positive feedback. Minor suggestions were made to improve the storyboard, including allowing flexibility for guests who may have personally experienced attitude change themselves, balancing narration and guest interviews, providing opportunities for reflective questions or activities, and ensuring that “outros” (final comments concluding the episode) are not rushed. A simplified version of this storyboard is presented in Figure 3.

I’m quite proud that that is a structure that’s been come up with and that there were so many inclusions in it of conversations have been had. It’s really, really exciting.

Committee member, female, age 42 years

#### Content Principles

In between focus groups 2 and 3, the members were asked in Slack to share examples of podcast logos, art, or graphics that they liked and why they liked them. On the basis of this information and general feedback received through previous focus groups and the survey in part 1.1, the lead facilitator prepopulated a Mural canvas that was based on a “world café” activity that facilitates intimate, small group conversations around certain prompts [43]. Each “table” on the canvas presented an aspect of the podcast related to marketing and content, including the logo, episode descriptions, episode topics, key messages, and podcast tone. Content principles were provided for each aspect, with sticky notes for members to provide feedback. In focus group 3, the participants moved around each virtual table in small groups and provided rapid feedback.

In the whole group discussion, the participants felt that the tone of the episode was important, emphasizing on ensuring that the tone is sincere, empathetic, and hopeful through the use of language and music. They suggested a range of potential episode topics, including employment, health care, mass media, education, housing, and legal services. The members had mixed views on the importance of creative elements, such as logos, graphic design, and music, although they agreed that these elements would contribute to the “tone” of the podcast. Generally, they showed a preference for clean and eye-catching imagery with clear and large text that avoided elements overused in general and in psychology or mental health podcasts (such as microphones, sound waves, or brains).

Through discussion, it was identified that episode descriptions should be short and punchy, clearly conveying what to expect in each episode. Hypothetical questions, dot points, bold text, and conversational language can be used to improve readability and draw in readers. The members also felt that episode messages should be clear, conveying the prevalence and impact of stigma and discrimination in each context with an intersectional lens. The messages can also revolve around challenging myths and stereotypes, informing the listener that they have a role to play within their sphere of influence in terms of their attitudes and behaviors. The members felt that it was important to acknowledge structural issues clearly while ensuring that listeners did not feel overwhelmed:

…Making sure that we do drive home the importance of the structural change as well...I mean, [listeners are] not necessarily gonna be able to set fire to the entire government and world, and start again, but some meaningful way that they can influence change at a structural level, not just within their immediate sphere.

Committee member, male, age 32 years

The members were advised that information from the world café activity, alongside the key decisions from previous focus groups, would directly inform how the podcast is produced after the study. The members were positive about the decisions that had been made because of the co-design process:

It feels like [the podcast is] in a good place, and it has sort of a launching pad to platform off now...we don’t know the specifics of how the episode is going to sound or your interviews, but I think it’s a really detailed, well thought out plan...it’s taken on everyone’s perspectives. Whilst not every idea could go through, but it’s been really refined. And I think it’ll be an amazing final product.

Committee member, female, age 25 years

## Discussion

### Principal Findings

This is the first known study to apply a co-design methodology to inform the design of a podcast that primarily aims to reduce stigmatizing attitudes toward people living with complex mental health issues. Across a 2-part co-design study, target audience members collaborated to inform the key features of the podcast: (1) individual episodes focusing on areas where stigma and discrimination are known to occur; (2) episode storyboards, including narratives from guests with lived experience, alongside those from academics, advocates, clinicians, and others; and (3) content principles, including sincere, empathetic, and hopeful tone; clear messaging; and calls to action.

The proposed podcast’s narrative-based design can be classified as both an educational intervention—as it will convey facts, trends, and recommendations—and a contact-based intervention, as it will share real stories directly from people with lived experience of complex mental health issues [44]. As seen in the elaboration likelihood model [45], educational elements have the potential to impact listeners through the *central* routes of processing, whereas the use of emotion, tone, music, and even moments of lightness or humor can impact the *peripheral* route. In this study, the participants felt that the podcast should raise awareness of the prevalence and impact of stigma and discrimination and that emotion would be integral to influencing listeners’ attitudes by increasing empathy during the listening process [46]. For example, the podcast may humanize people living with mental health issues, surprising or angering listeners as they learn of injustices, encouraging listeners to understand and relate to the emotional impact of discrimination, and potentially driving listeners to reflect on their own views and behaviors. As stated by Shen et al [11], “narratives have the unique ability to involve audiences mentally by transporting them into the narrative world and arousing emotional reactions.” Indeed, previous research has found that many elements of the newly designed podcast—such as the explicit expression of emotions and emphasis on struggles and challenges—are empathy arousing and can lead to a reduction in social stigma [47]. Furthermore, the linear storytelling structure is likely to result in a higher level of suspense, which is associated with higher levels of engagement and, therefore, persuasion [48].

The podcast design also aligns with recommendations for future stigma reduction directions, as identified by Walsh and Foster [6]. They argue for contextualizing stigma messaging to increase generalizability and reach audiences more directly, that is, weaving together the physical, social, and organizational environments in which the public understandings of mental health issues are formed. By focusing each episode on domains in which stigma and discrimination are known to occur, there is an opportunity to do this. Furthermore, the new podcast aims to centralize people with lived experience in each episode, which also aligns with their recommendations. In addition, the committee recommended minimizing reference to the medical model. This aligns with research that has found that focusing on the biogenetic and neurochemical contributors to mental health issues can unintentionally perpetuate stigma [6]. However, guests and listeners who may be familiar with the medical model should not be isolated or denigrated. All guests will be encouraged to use their preferred language during recording, with an opportunity to ask questions and discuss, and the narration will be scripted to ensure that language is used in a balanced manner.

Many findings in this co-design study also align with the previous literature around podcast development, for example, research highlighting the importance of providing good-quality and accurate content, maximizing accessibility [26,49], and ensuring that the podcast listeners know what to expect in each episode to facilitate informed consent before listening [13]. The proposed podcast will involve clear signposting and content warnings, share a variety of lived experience perspectives alongside academic or clinical knowledge, and provide transcripts. However, some findings contrasted with previous recommendations; in particular, previous studies recommended academic rigor and a professional tone as opposed to a more entertaining or conversational approach, albeit in the medical education context [26,49]. In this study, which was predominantly about attitude change rather than increasing mental health literacy, it is fair to assume that a different approach is needed to ensure an impactful podcast.

This study offers new insights into an effective co-design methodology for developing a podcast. The decision to start part 2—co-design—with the topic of each episode’s focus and then move to more narrow and specific decisions was effective. Importantly, the committee worked together very effectively and respectfully. This was in part because of careful planning and communication from the researchers to minimize the impacts of common challenges to co-design, such as uneven power dynamics, dominant voices, or tokenistic involvement [4,37]. Feedback was very positive (Multimedia Appendix 5), and the participants praised the facilitation of the group, saying that they felt safe and that their voices and opinions were genuinely heard. Feedback on the use of technology in the co-design committee, particularly Mural and Slack, was also positive. The facilitators were also responsive to feedback about time management and the structure of the discussion to minimize fatigue.

Similar to the study by Smith et al [32], there were some tensions in focus groups between telling genuine stories and producing a suitable podcast; indeed, the committee felt that a podcast that focused solely on storytelling or raising awareness could be ineffective at reducing stigma or risk perpetuating stigma. There was a strong desire from the participants with lived experience to raise awareness of the reality of the prevalence and impact of stigma and discrimination and to provide more of a thoughtful and critical lens to how people develop stigmatizing attitudes and how systems and structures perpetuate discrimination. Again, these concerns have been highlighted by previous researchers who argue that contact-based interventions should not assume that the public are unfamiliar with mental health issues but rather consider how the public continue to see people experiencing mental health issues as “other” despite proximity and familiarity [6]. The discussion identified that such content was important but could leave individual listeners overwhelmed and unsure about their power to reduce structural stigma and discrimination. Eventually, the committee settled on strategies for balancing this risk, including having clear calls to action that focused on individual attitudes and behavior changes. A key tension will be ensuring that while the podcast draws attention to instances of stigma and discrimination, it does not result in a “backfire effect”—unintentionally reinforcing listeners’ stigmatizing attitudes. This has been observed in response to “myths versus facts” messaging campaigns, such as increasing the perceptions of danger (eg, Dobson and Rose [50]), but its applicability to the current context is currently unknown.

The co-design process also unearthed potential challenges associated with the podcast. Although most podcast listeners in part 1.1 would considering listening to a new podcast about stigma and discrimination, such framing may be off-putting for some listeners. In addition, although the podcast aims to affect both attitudes and behaviors, it is more likely to affect the shorter term outcome of attitude change rather than longer-term behavior change [5]. To maximize both the listenership and the impact of listening, the committee identified strategies such as contextual focus of episodes, marketing the podcast in terms of improving outcomes (rather than being explicit about stigma), and ensuring that the tone and marketing highlight optimistic and empowering messages while ultimately raising awareness. Ideally, both naive and informed listeners should be able to learn something from the podcast.

### Limitations

Like many co-design studies, this study was adapted from existing co-design methodologies rather than using an established methodological framework in a rigid way. This was necessary because of the novel nature of the study, but future studies may wish to compare and contrast methodologies to determine which co-design activities are best suited to co-designing podcasts. Furthermore, in many situations, coproduction, which involves stakeholders having a more active role in service or product design and implementing a solution, is preferable to co-design [1]. However, owing to time and funding constraints, participatory involvement could not extend into production of the podcast itself, such as reviewing prototypes [33]. The researchers intend to evaluate the impact of the podcast in a future study, which will allow an opportunity for listeners to provide feedback before any kind of public launch.

Although the focus groups ran well, there were some challenges, such as those seen in previous studies (eg, Slattery et al [4] and Dimopoulos-Bick et al [37]). Although key target audiences were represented, there were fewer expressions of interest than anticipated and little ethnic diversity in the focus groups, limiting generalizability. There were minor issues with the web-based setting, such as internet connection issues and a learning curve with Mural. There was also some attrition in the co-design committee. Only 6 (60%) of 10 members attended the final focus group, despite the request that prospective members only join the committee if they were sure that they could commit to all focus groups. The reasons for not attending the final focus group included unexpected work and family commitments (as some Australian COVID-19 restrictions eased in the middle of the study) or becoming unwell and needing to access medical care. Similar challenges have been reported in other studies [38]. Of the 10 committee members, only 1 (10%) stopped attending without an explanation and could not be contacted. Attendance may have also been impacted by screen fatigue or dissatisfaction with the group, which was not captured in the optional feedback surveys. Although attrition leads to risks of imbalanced representation and a lack of input [37], all target audiences were still represented in the final focus group, reducing this risk.

There are some limitations around the analysis of qualitative data. Of note, the initial analysis of the open-text responses in part 1.1 to draft touch points was conducted by only 1 researcher, and this was an informal process owing to timing constraints. Of note, previous literature has reported a variety of analytical approaches, and there is no specific analytical approach recommended [36]. It is possible that the subjective nature of this analysis led to the identification of touch points that did not wholly reflect the audience members’ priorities for future co-design work. Owing to resourcing constraints, there was no capacity for member checking of the transcripts or the analysis conducted in part 1.2; however, the co-design committee was highly active and vocal about the areas that mattered the most to them, which validated the touch points drafted and refined in part 1.

### Conclusions

Through co-design, the target audience members identified a range of strengths and limitations of a podcast that aims to reduce stigmatizing attitudes and discriminatory behaviors toward people living with complex mental health issues. The co-design committee members worked well together to inform the proposed podcast’s focus, storyboards, and content principles. The new podcast will be produced by the research team. It is intended that the podcast will be launched to the public after an evaluation study, made available on a podcast streaming service, and promoted by collaborating organizations. Future research should further explore how co-design processes can be adapted to podcast development, explore different types of podcast design for stigma reduction and attitude change, and identify how other stigma reduction interventions can use popular media in innovative ways.

## Appendix

Multimedia Appendix 1 Participants’ preferences for a new podcast on the topic of mental illness stigma.

Multimedia Appendix 2 Information gathering focus groups—agenda.

Multimedia Appendix 3 Information gathering focus groups—feedback.

Multimedia Appendix 4 Co-design focus groups—agenda.

Multimedia Appendix 5 Co-design focus groups—feedback.

## Abbreviations

CASPR: Co-Design and Anti-Stigma Podcast Research
EBCD: Experience-Based Co-Design

## Data Availability

The data sets generated during this study are not publicly available to protect the participants’ privacy but are available from the corresponding author upon reasonable request.
