# Supplementary figures and images for: A New Podcast for Reducing Stigma Against People Living With Complex Mental Health Issues: Co-design Study
Source: JMIR Form Res. 2023 May 5;7:e44412. doi: 10.2196/44412 (PMC10199394; doi:10.2196/44412)

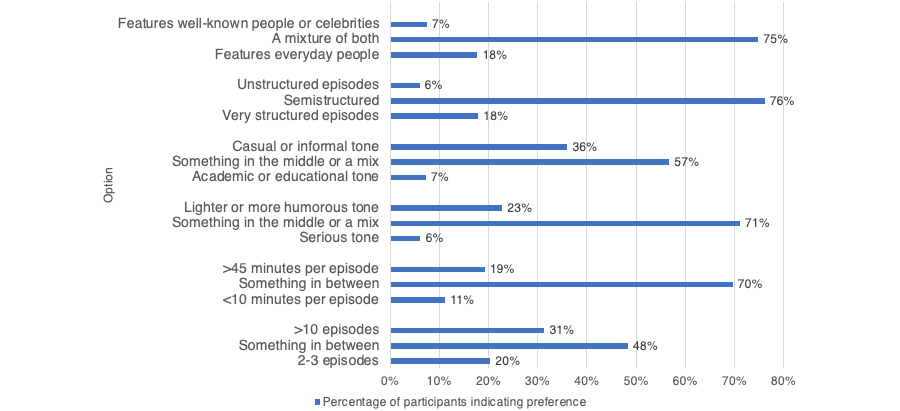

Supplement: Multimedia Appendix 1 [file formative_v7i1e44412_app1.png]
