# Supplementary material for: A New Podcast for Reducing Stigma Against People Living With Complex Mental Health Issues: Co-design Study
Source: JMIR Form Res. 2023 May 5;7:e44412. doi: 10.2196/44412 (PMC10199394; doi:10.2196/44412)
Supplement: Multimedia Appendix 2 [file formative_v7i1e44412_app2.docx]

Multimedia Appendix 2

# Information Gathering Focus Groups – Agenda

| **5pm**  **Introduction and housekeeping** | **Aims:** *This activity aims to ensure that:*   - *Participants understand the scope and objectives of the focus group* - *Participants understand how their psychological safety and wellbeing will be protected and promoted throughout the focus group, including the supports available to them if they feel distressed or unsafe*   **Intro:**  Lead Facilitator:   - Welcome participants - Acknowledgement of Country - Introduce herself and her background/role - Live transcript – inform participants how to turn on Zoom live transcript function if wanting captions   Support Facilitator (Rotating)   - Introduce themselves - Describe their role (i.e. to support participants if they need support, message via Zoom private chat if wanting to touch base)   **Icebreaker:**   - All participants to introduce themselves by first name, and mention - *What state are they dialling in from?* - *What is your favourite rainy-day activity?* (Or other ice breaker question)   Lead Facilitator to share **introductory PowerPoint slides**   - Present agenda for today’s session: introduction, group discussion, collaborative group activity - Explain why we want to work with [target audience group] - Group guidelines/Facilitator to provide **group guidelines** (see below)   - Any questions? Anything people want to add? - Facilitator to discuss what to do if needing to take a break, having technical difficulties or if internet connection is unstable: e.g. - Turn video off - Leave and re-join session (use Portable Hotspot if needed) - Dial in if needed - Please directly message or email one of the Facilitators if having ongoing trouble - Please directly message the Support Facilitator if you want to chat separately - If facilitators experience internet problems, will set up hotspots – give us a few minutes and sit tight! - Encourage use of Raise Hand feature - Chat will be saved - Discuss scope of project – Confirmed: podcast itself, aims of podcast, target audience will feature guests with lived experience. Not yet confirmed: tone, number of episodes, episode content, how we share stories, marketing, and framing - Any questions? |  |
| --- | --- | --- |
| **5:30pm**  **Group discussion of touch points** | **Aims:** *This activity aims to gather information about participants’ levels of agreement and disagreement with findings from Part 1.2 as well as existing stigma reduction literature.*  Facilitator to turn on recording and note that breakout rooms will not be recorded.  **Actions:**  Lead Facilitator presents a series of neutral statements and asked to discuss them with the group. These are related to a series of ‘touch points.’  Neutral statements for discussion (aim to present at least 2, ideally 3, depending on time and nature of discussion):   1. *People may not listen if they don't think it is relevant to them - despite holding stigmatising attitudes themselves.* 2. *Telling real stories may (or may not) be enough to change listeners' attitudes.* 3. *A podcast about stigma and discrimination could be emotional to listeners* 4. *People working demanding jobs may not wish to listen to a podcast on a serious topic.* 5. *It is difficult to represent everyone’s story in a podcast.*   Examples of discussion questions:   - Do you agree or disagree with the statements? - Why do you agree or disagree? - How do you interpret this statement? - How does this statement apply to your work/school/experience?   **Pause recording** |  |
| **6:15pm at latest**  **Break** | Approx. 10 minutes | |
| **6:30pm at latest**  **Empathy map activity**  **6:55pm** | **Aims:** *To build on the previous activity, reflecting on how the touch points may interact and relate to target audiences, and encourage participants to bring forth new ideas not already discussed.*  **Link:** [Mural link provided on night]  **Actions:**  Lead Facilitator to present:   - An **empathy map** is a collaborative activity that helps us understand what different people think, feel, and do. In this case, I want to understand what podcast listeners representing [target audience] may think, feel, and do - Will inform the development of the podcast to make it as strong and useful as possible - *Brief Mural.co navigation overview. Quick tips: Double click to add a sticky note. Scroll to zoom in/out or use controls on bottom right of screen*. *Click and drag to move.* - Ask participants to collaborate and determine general behaviours, attitudes, needs, motivations in relation to a podcast on the topic of stigma and discrimination. Some of these might have been already discussed with the wider group and can be re-used/expanded on - Note: don’t stress too much about what goes where, or capturing every person’s experience. No right and wrong answers - Group 1 (attended by Lead Facilitator) to work on ‘say’ and ‘do’ (purple/left). Group 2 (attended by Support Facilitator) to work on ‘think’ and ‘feel’ (blue/right) - Will ask a group member to summarise when back in the group   Lead Facilitator splits group into two breakout rooms. One facilitator will observe each group and act as scribe, sharing their screen.  After about 20 minutes, Lead Facilitator ends the Breakout Room activity.  **Turn on recording**  A representative from each group will then present their half of the empathy map with the wider group for discussion. Potential discussion questions:   - What were some of the key themes that came up in your discussion? - Was there anything you struggled with? - What do you think are the most important things to keep in mind for the podcast development? | |
| **7:20pm**  **Wrap up/final thoughts** | Depending on time, Lead Facilitator will ask:   - Does anyone have any final reflections to share?   Lead Facilitator to send link to Feedback Form and note that expressions of interest are open the Co-Design Committee (as part of Feedback Form).  **Link to feedback form**: [Qualtrics link provided on night and emailed] | |
