# Supplementary material for: A New Podcast for Reducing Stigma Against People Living With Complex Mental Health Issues: Co-design Study
Source: JMIR Form Res. 2023 May 5;7:e44412. doi: 10.2196/44412 (PMC10199394; doi:10.2196/44412)
Supplement: Multimedia Appendix 3 [file formative_v7i1e44412_app3.docx]

Multimedia Appendix 3

# Information Gathering Focus Groups – Feedback

Twenty-one of the 25 participants completed the feedback. Feedback was positive across the three questions, which had a maximum score of 5 indicating ‘strongly agree’. Twenty of the 21 respondents indicated interest in joining the Co-Design Committee. Open-text feedback was generally positive, with participants commenting the conversation being ‘stimulating’ and ‘respectful’, with a ‘good mix of professions and backgrounds.’ Suggestions for improvement included having larger groups, a slightly longer break, and more interactive activities.

Information Gathering Focus Group feedback (Aggregated across all four focus groups)

| Question | *M* | *SD* |
| --- | --- | --- |
| I felt that my voice was heard during the focus group | 4.8 | 0.4 |
| I felt safe during today’s focus group | 4.8 | 0.4 |
| The focus group was engaging | 4.7 | 0.5 |
