# Supplementary material for: A New Podcast for Reducing Stigma Against People Living With Complex Mental Health Issues: Co-design Study
Source: JMIR Form Res. 2023 May 5;7:e44412. doi: 10.2196/44412 (PMC10199394; doi:10.2196/44412)
Supplement: Multimedia Appendix 4 [file formative_v7i1e44412_app4.docx]

Multimedia Appendix 4

# Co-Design Focus Groups – Agendas

## Co-Design Committee Meeting 1

| **Introduction**  **10 minutes** | ***Aims:*** *This activity aims to ensure that:*   - *Participants understand the scope and objectives of the Co-Design Committee and its focus groups* - *Participants understand how their psychological safety and wellbeing will be protected and promoted throughout the focus group, including the supports available to them if they feel distressed or unsafe* - *Participants understand the expected commitment and engagement with the Co-Design Committee and value others’ expertise, and start to feel settled and welcome*   **Actions:**   - Lead Facilitator will welcome participants, provide Acknowledgement of Country, and introduces Support Facilitator - Support Facilitator to introduce himself and describe his role (i.e. to support participants if they need support) but also will be involved in this discussion in a supporting role - Facilitator will confirm the agenda and purpose of today’s session and what to expect:   - Introduction to co-design, and discuss working together effectively  - Group activity to start deciding on elements of the podcast  - Remind participants that this is both a research and co-design process - request participants are mindful of their privacy and others’ privacy and avoid sharing surnames, workplace names or personal details |
| --- | --- |
| **Icebreaker**  **10 minutes** | - All participants to introduce themselves by first name, and mention:   *a) which focus group did you attend initially?*  *And b) what is your favourite breakfast food? (icebreaker)* |
| **Background**  **5 minutes** | Lead Facilitator to **turn on recording** and share screen, and note that breakout rooms will not be recorded.   - Facilitator to reiterate aim, background of study - Summarise study findings so far including findings from Part 1.1 and 1.2 |
| **What is co-design? And working together**  **10 minutes** | What is co-design?   - Present some key information about co-design: *A democratic process whereby end-users, and other key people, are involved in a meaningful way in design processes*   Process considerations   - We are a **mixed group** - everyone has similar interests and cares about this topic, but we all come from different backgrounds and many of us identify with different target audiences - For example, some of us may have unintentionally been discriminatory towards people with lived experience, or work in settings or systems that do not meet the needs of people with lived experience of mental health issues - Sometimes we use language that we resonate with, but others do not - We don’t have to shy around difficult topics, but I ask everyone to keep these challenges in mind - This isn’t co-production or consumer-owned/led – meaning Committee will contribute towards *some* but not all decisions. This is a limitation of our design and based on resources, timing, staff etc. Committee will not be able to provide feedback on the final product due to the study’s resourcing   **Group guidelines**   - Present and discuss group guidelines (below) - Also: it’s ok to turn your camera off if you need to, leave early, or withdraw, but – if possible – please send Support Facilitator a message just to let us know - Remember we will be using Slack – more of an opportunity to share things outside of sessions - The Support Facilitator here if you want to talk to someone during the group, but you are welcome to talk with the Lead Facilitator outside of this group – we can set aside time to chat about any concerns you might have. - Opportunity for feedback at the end.   **Stop sharing slides & ask group:**   - If you have worked in a co-design group before – or anything similar like this, what were some of challenges? What were some of the benefits? |
| **Break**  **10 minutes** | **Pause recording** |
| **Group discussion of first touchpoint**  **50 minutes** | ***Aims:*** *To orient participants to the first touch point to be discussed throughout the Co-Design Focus Groups and to begin to generate ideas for addressing the challenges and opportunities identified in previous focus groups*  ***Activity:***  *Lead Facilitator to present the first touch point to be discussed throughout the Co-Design process.*  **Restart recording** and share screen  **Scope: What is confirmed/ what isn’t**   - Confirmed: podcast itself, aims of podcast, target audience, will feature guests with lived experience. Expect listeners to have an emotional response to the podcast including empathy and hope. Expect some plain language educational messages/calls to action, linking to resources. Small budget for production phase + transcription. - Not yet confirmed: tone, number of episodes, episode content, how we share stories, marketing and framing, exact messages or calls to action, creative elements (music, art, logo), how explicitly we will focus on stigma/discrimination - Any questions?   **Touchpoint 1**   - Today’s focus: ‘Listening to and engaging with episodes’ - Questions we hope to eventually answer include: What is the story we want to tell? And how do we tell a meaningful and engaging story? What is it that we want to focus on at the episode level?   **What we want to discuss first:**   - What **focus** is best for individual episodes? - Why are we starting here: this is a central aspect of podcast production, and will impact a lot of areas. This will begin to inform other areas, such as key messages, how we expect the podcast to impact on listeners, and who is most likely to listen - Note that decision-making in co-design is rarely linear so we might jump around - Present five initial options for episode foci:   1. Individual LE Story: An **individual’s experience living with mental health issues**, and/or their experience with recovery   2. Individual Stigma Story: An **individual’s experience with stigma and discrimination** (more explicitly than their overall experiences)   3. Mental Health diagnosis/experience Focus: A **focus on mental health specifically** – e.g. diagnosis-focused, or aspect focused (e.g. educate around that experience/disorder, challenge stereotypes)   4. Stigma Area of Concern: An **area or domain where stigma and discrimination is likely to occur** (e.g. relationships, workplaces, healthcare, mass media, police interactions)   5. Stigma Concept deep dive: **Deeper dive into an aspect of stigma and discrimination** (e.g. stereotypes and myths, prejudice, discrimination, public stigma, self-stigma) - Any questions/clarifications around these options?   **6:30pm**  Lead Facilitator to share screen with Mural activity, and orient participants.   - Link: [provided on night] - Remind everyone on how to use Mural – how to zoom in, move the canvas, and type in sticky notes - Splitting into 5 breakout rooms with 2 people each – Facilitators to drop in. - Everyone to answer question prompts including: strengths and limitations of each option, who would be likely to listen, potential messages and emotional impact - Will allow approx. 20 minutes. - Ask someone to report back to group   **Breakout rooms**  After 20 minutes, bring back everyone and **turn on recording**   - Groups to report back: (~3 mins each, so up to 15 minutes) – observations from each option |
| **Wrap up/final thoughts** | **7:20 pm** at latest  Depending on time, Lead Facilitator will ask: Does anyone have any final reflections to share?   - Mention feedback link and Slack invitation will be emailed ASAP - Please join Slack this week – Lead Facilitator will post some question prompts - Remind participants of date/time of next focus group - Will start to organise payments. Send bank details if haven’t already   **To email after focus group:** feedback link, invitation to join Slack and information on how to use Slack. |

## Co-Design Committee Meeting 2

| **Introduction**  **10 minutes** | ***Aims:*** *This activity aims to ensure that:*   - *We check in/feel settled*   **Actions:**   - Lead Facilitator will welcome participants, ask how everyone has been/how we’re all tracking - Acknowledge any absences - Icebreaker: *“What is something that has made you happy this past fortnight?”* - Lead Facilitator will confirm the agenda and purpose of today’s session and what to expect: - Group activity to reach consensus on the focus of each episode - Moving on to episode structure and storyboarding - If have time will start to workshop some key messages/calls to action as part of the storyboard (informing touch point: ‘Translation to attitude change and action’) |
| --- | --- |
| **Focus of episode**  **50 minutes** | **Turn on recording**  ***Aims:*** *To discuss and eventually reach consensus around the focus of each episode, in order to move on to the next activity (storyboarding), and ensure that group members who may not have used Slack in the past fortnight are able to catch up on discussions*   - Ask group: Any thoughts/reflections/feedback about Slack so far?   Lead Facilitator to update group on Slack discussion:   - Getting the strong sense that people prefer including multiple perspectives and voices, in some capacity - We've discussed the value of including authentic lived experience stories, as well as bigger picture discussions about stigma and discrimination - Ideas have been raised to have more of a flexible or semi-structured approach rather than having a pre-defined focus for each episode - An idea being workshopped in Slack is to start with a guest with lived experience sharing their story, then do a deeper dive into a key aspect such as the context where that person has experienced stigma + discrimination (e.g. workplace, educational setting) - Providing context in the marketing + episode description may help listeners decide to tune in and give the episode a try.   Lead Facilitator to share screen and present iterations:   - Based on the group discussion, Slack discussion, and previous research, I have reworked this to two options – these are more ‘loose’ options for the episode focus but are both ‘hybrids’ of the existing options  1. **Hybrid: Individual lived experience focus**  - Focuses on **one** **individual’s** experience living with mental health issues, and highlights their experiences with stigma and discrimination - Podcast episode may then expand on themes brought up on their story – e.g. interview an advocate, academic or clinician about the topic, key terminology, or statistics, what needs to change, etc - Example episode titles: - *Joe: Flexible work & workplace rights* - *Mei: Culture & self-stigma* - *Kate: Being a doctor with lived experience*   **2. Hybrid: Context focus**   - Context-first – themed around an area or domain where stigma and discrimination is likely to occur, like workplaces, healthcare settings, media - Features stories from multiple people with lived experience, and potentially brings in advocates, academics, or clinicians to provide insight - Example episodes: - *How can workplaces support people living with mental health issues?* - *When your culture doesn’t talk about mental health* - *Doctors can be unwell, too* - Support Facilitator to access Mural and share screen, zoom in on **prioritisation matrix.** Axes labelled: ‘Impactful’ and ‘Engaging’   Link: [provided on night]  Open group discussion for each option:   - Considering the general audience, how engaging do you think this option would be? - And how much do you think this option could impact on listeners, in terms of changing their thoughts, attitudes or even their behaviours? - Rate each on prioritisation matrix   If group has not reached consensus, or if items are rated equally impactful/engaging, use Zoom poll for Committee members to anonymously choose their preferred option. |
| **Break**  **10 minutes** | **Pause recording** |
| **Podcast episode structure discussion**  **10 minutes** | **Turn on recording**  ***Aims:*** *To present options for different types of podcast structures and storytelling devices.*  Lead Facilitator to share screen and present some broad options for content to guide thinking   - Clips from interviews with different people (people with lived experience, advocates, policymakers, academics, clinicians, etc.) – can be lightly edited or ‘woven’ through an episode - Panel-style discussions - Narration by a host(s) - Music (to a degree) - Sound effects (to a degree) - Clips taken from other media (within copyright/fair use)   Lead Facilitator to discuss common ‘three-act’ structure of storytelling: setup, confrontation, and resolution:   - The three-act podcast structure is a storytelling model that divides a story into an organized and logical order. - This is a *rough* structure that we know is effective. Of course, people’s experiences don’t always fit this structure, but you would be amazed how often this structure is seen in media, even in interview or solo podcasts - *Setup*: establish a setting, introduce key people, set up the stakes, hooking listeners - *Confrontation*: raise the stakes, explore real challenges, talk about attempts to solve the problem, ups and downs - *Resolution*: provide closure, solve problems and/or bestow lessons – what do you want listeners to walk away with?   Lead Facilitator to present listeners’ preferences for a podcast on the topic of stigma from Part 1.2   - Participants preferred a mix of guest types, semi-structured episodes, a casual or mix of tones, a mix of seriousness and humour, a length between 10-45 minutes (let’s aim for 15-30 mins)   Ask group:   - What are your thoughts around these different options? - Which options might be appropriate for the current podcast, and why? |

| **Storyboard**  **45 minutes** | **6:35pm**  ***Aims:*** *To gain ideas for what to include in individual episodes via a rough storyboard. This will then be refined and presented back to the group for feedback in the final focus group*  Lead Facilitator to present Mural storyboard activity – template with six ‘scenes’ for the podcast episode, from Setup/Introduction to Resolution/Wrap Up.   - Recommend keep storytelling structure in mind - Use sticky notes and text to add different steps. Can also add images or gifs if you are comfortable using Mural (see left hand side) - Plan is that I will look at these and workshop in between, and will present for feedback next session. - Will break into two groups to create own storyboards: Group 1 to work on example episode around workplaces, Group 2 to work on example episode around educational settings. - A Facilitator will act as scribe for each group   Link: [provided on night for each breakout room]  **Breakout room activity**  **Turn on recording when returning to main discussion**   - Ask group if there is anything they wish to highlight from their storyboard? |
| --- | --- |
| **Wrap up/final thoughts**  **5 minutes** | Depending on time, Lead Facilitator will ask: Does anyone have any final reflections to share?   - Remind group to provide feedback and check Slack - Remind participants of next meeting date/time   **To email after focus group:** Feedback form |

## Co-Design Committee Meeting 3

| **Introduction**  **10 minutes** | ***Aims:*** *This activity aims to ensure that:*   - *We check in /feel settled*   **Actions:**   - Lead Facilitator to welcome participants, ask how everyone has been/how we’re all tracking - Acknowledge any absences - Icebreaker: *“What is something that you’re looking forward to in the next few weeks?”* - Lead Facilitator will confirm the agenda and purpose of today’s session and what to expect:  1. Reviewing updated storyboard – seeking feedback 2. Content principle/style guide activity – seeking rapid insights around a few areas 3. Reflect + celebrate what we achieved |
| --- | --- |
| **Storyboard discussion and feedback**  **40 minutes** | **Turn on recording**  Lead Facilitator to share screen with storyboard:   - Based on the group discussion and Slack discussion, I have created a combined version of the two storyboards - This storyboard starts with a ‘hook’ (quote), involves a narrator who introduces the topic and links sections together. Guest(s) with lived experience are introduced and they discuss their experiences with stigma and discrimination in the areas of interest. Additional subject matter experts may be included to discuss research, best practice, or initiatives in this area. Episodes end with calls to action (actions listeners can take themselves), resources and credits) - The storyboard is semi-structured – so the idea is, we would keep the broad format, but things might shift around slightly in terms of order or depth of content. - And it may be adapted depending on the content of interviews in the future + who is available   Ask group:   - What are your overall thoughts around this storyboard? - What are its strengths? - Is anything missing? |
| **Break**  **10 minutes** | **Pause recording** |
| **Content principle activity**  **45 minutes** | **Turn on recording**  ***Aims:*** *To obtain rapid feedback around ideas for marketing and framing the podcast, and general content principles. This will relate primarily to two touch points: marketing and framing the podcast, and translation to attitude change and action.*   - Lead Facilitator to share screen with Mural activity: Each ‘table’ involves an aspect of the podcast related to marketing and content, including logo, episode descriptions, episode topics, key messages, and podcast tone and language. Draft content principles are included for each aspect, with sticky notes for participants to provide feedback. - Note that these content principles were developed based on a) discussion in the focus groups, b) findings from the survey I spoke about last week, c) Slack discussion, and d) recommendations from the literature - Note that unfortunately, we don’t have creative elements like logos or music to workshop yet, but the feedback from today will inform the choices we make. - Two small groups of 3-4 people to move between different ‘tables’ - About getting **rapid feedback**. Task is to spend approx. 5 mins per ‘table’ – 30 minutes for this activity – prompt question is ‘Does this sound right to you?’ and ‘How might we strengthen this area?’. Small groups to discuss different options, make notes or suggestions if you have any. If you think it’s right, make a note of that! - Some tables will need more discussion than others and not everyone may get to every table - A facilitator to be scribe for each group. Short notes are fine – we will discuss as a group. 2 groups: Group 1 start at table 1 (title + logo), Group 2 start at Table 3 (individual episode topics) - Link: [provided on night]   **Breakout room activity**  **Turn on recording when back in main discussion**   - Ask group about overall impressions of content principles - Anything they wish to highlight? |
| **Reflection activity and celebration**  **15 minutes** | *Aims: To reflect on what we’ve achieved as a group, the decisions we’ve made, and celebrate*  Lead Facilitator to summarise verbally what we’ve achieved   - Worked together respectfully and effectively - Identified the focus of episodes – initial options weren’t quite right, but we were able to workshop these and land on an option that has potential to be engaging and impactful - Identified many creative ideas for episode content and determined a storyboard - Informed overall approach, language, design, tone, etc   Open discussion:   - What have been the highlights? - How are you feeling about the podcast at this stage? - There will be an opportunity for feedback on the process through the survey link – but does anyone want to discuss feedback verbally about the co-design process? |
| **Wrap up/final thoughts**  **5 minutes** | - Thank everyone for their involvement - Slack to be archived 2 weeks after final focus group - Will send email summary after the data are analysed and the plans are set for production, and we will let everyone know about what happens next - especially if things go well and we launch publicly   **To email after focus group:** Feedback form and thank you message |

## Focus Group Values

Summary of group guidelines discussed in Information Gathering and Co-Design Focus Groups

| Value | Key messages |
| --- | --- |
| *Collaborate in an open and cooperative manner* | The podcast can’t cover everything, and we can’t expect it to. But we can work together to figure out the best options to make it effective, be open to ideas, and work together to incorporate feedback. |
| *Respect all forms of expertise* | We all bring something to the table. Whether this is personal experience, research or clinical expertise, expert in communications, academic research, et cetera. It’s important to give everyone a chance to speak. We will have different views; disagreeing is welcome, and normal. |
| *Privacy* | Respect and safeguard each other’s privacy. What is said in the group should stay in the group, unless there are safety concerns that come up. Please do not share information about other Committee members outside of this group. |
| *Safe sharing* | Stigma can be a difficult topic. Sharing is welcome, but please be mindful of what you say, especially when discussing any personal experiences of mental ill-health or discrimination. Consider how stories might affect yourself and others. Sometimes it is helpful to share more ‘general’ rather than ‘specific’ examples. |
| *Communicate openly and honestly* | Please keep in touch with the facilitators about your thoughts, availability, or requests. This includes if you need to send an apology for a meeting, or withdraw from the study. We are happy to discuss flexible options including how we can help you participate. |
