# Supplementary material for: A New Podcast for Reducing Stigma Against People Living With Complex Mental Health Issues: Co-design Study
Source: JMIR Form Res. 2023 May 5;7:e44412. doi: 10.2196/44412 (PMC10199394; doi:10.2196/44412)
Supplement: Multimedia Appendix 5 [file formative_v7i1e44412_app5.docx]

Multimedia Appendix 5

# Co-Design Committee – Feedback

The optional feedback form was completed on 16 occasions across the three Co-Design focus groups, with 9 of the 10 participants completing the form at least once. Feedback was highly positive across the three Likert quantitative feedback questions, which had a maximum score of 5 indicating ‘strongly agree’ (see Table 4, below).

In written feedback for the first focus group, comments were mostly positive, though several participants commented on the importance of time management; this focus group ran slightly over time and some of the activities were somewhat rushed. One participant commented on the need to ‘remain agile’ based on feedback. However, this focus group’s discussion was continued in Slack and picked up in the next focus group, and the facilitator was able to modify options based on feedback.

Comments on the latter focus groups were very positive, including the conversation being ‘interesting’, ‘respectful and insightful’, and that participants ‘loved hearing everyone’s different perspectives.’ Latter focus groups were praised for the facilitation, and the agenda being ‘really well balanced.’ By the end of the focus groups, participants reflected on the process, with comments around the group being ‘motivated and positive’, and that their opinions felt valued. The use of technology was generally well received, with participants liking the use of Mural and Slack in particular, noting that the learning curve was manageable. Though, some commented on their preference for in-person or hybrid focus groups if possible, or a social event, if had been feasible with COVID-19 restrictions at the time.

Co-Design Committee feedback (aggregated across 3 focus groups)

| **Question** | ***M*** | ***SD*** |
| --- | --- | --- |
| I felt that my voice was heard during the focus group | 4.7 | 0.8 |
| I felt safe during today’s focus group | 4.7 | 0.3 |
| The focus group was engaging | 4.8 | 0.5 |
